# Hepatocellular carcinoma in Ghana: a retrospective analysis of a tertiary hospital data

**DOI:** 10.11604/pamj.2020.36.43.21085

**Published:** 2020-05-28

**Authors:** Kenneth Tachi, Adwoa Agyei-Nkansah, Timothy Archampong

**Affiliations:** 1 Department of Medicine, Medical School, College of Health Sciences, University of Ghana, Accra, Ghana

**Keywords:** Hepatitis, Korle Bu Teaching Hospital, surveillance, abdominal pain, weight loss

## Abstract

**Introduction:**

Hepatocellular carcinoma (HCC) is a cancer of global public health concern because of its high incidence and mortality. The impact is greatest in areas with high prevalence of its major risk factors including chronic hepatitis B virus (HBV). HBV is endemic in Ghana but a comprehensive data on HCC is lacking. The aim of this study was to describe the clinical, laboratory and radiological features of HCC at the Korle Bu Teaching Hospital in Ghana.

**Methods:**

The medical records of 194 HCC cases attended to at the Gastrointestinal Clinic of the Korle Bu Teaching Hospital between January 2015 and December 2018 were retrospectively analyzed for demographic, clinical, laboratory and radiological data.

**Results:**

The male: female ratio was 2:1 and mean age was 45.2 years. Weight loss and abdominal pain were the major presenting symptoms. No patients were identified through surveillance. HBsAg was positive in 109/145 (75.2%) of cases tested. Sixty-five (59.6%) of 109 HBsAg positives were aware of their HBsAg status but only 3 were receiving medical follow ups prior to the diagnosis of HCC. Raised alpha-fetoprotein level >165.2 IU/ML was found in 53.9%. One hundred and forty-four patients were eligible for only analgesia.

**Conclusion:**

HBV infection is the leading aetiologial risk factor associated with HCC. Majority of HBV carriers are aware of their status but do not receive care prior to HCC diagnosis. Majority present late and are eligible for only palliative treatment. Improvement in the health seeking behavior of HBV carriers can aid early detection of HCC.

## Introduction

Hepatocellular carcinoma (HCC) is the sixth most common cancer in the world, but its very poor prognosis makes it the third leading cause of cancer-related mortality worldwide [1]. Its epidemiology varies by region in parallel with prevalence of the major risk factors; chronic HBV and hepatitis C virus (HCV) infections [2]. More than 80% of HCC occur in developing economies, with Asian and sub-Saharan African (SSA) countries being the most afflicted [1]. In Asia and SSA, HBV is the most common risk factor for HCC, while HCV is the most common risk factor for HCC in North America, Europe, Japan and Egypt [1-3]. Ghana is a SSA country with a high prevalence of chronic HBV, estimated at 12.9% [4]. HCV is not uncommon at a prevalence of 3% [5]. The incidence and mortality rates of HCC in Ghana are estimated at 17.6 and 17.1 per 100,000 respectively [6]. HCC was also reported as first and third leading causes of cancer deaths in men and women respectively between 1991 and 2000 in Ghana [7]. Data on several other aspects of HCC is however lacking, resulting in a poor appreciation of the high burden of the disease and the impact of chronic viral hepatitis on the population. The aim of this study therefore was to comprehensively describe the clinical, laboratory and radiological features of HCC at the Korle Bu Teaching Hospital in Ghana.

## Methods

This study used a retrospective design to evaluate cases of HCC who attended the Gastrointestinal (GI) Clinic of the Korle Bu Teaching Hospital in Accra, Ghana between January 2015 and December 2018. The Korle Bu Teaching Hospital (KBTH) is a tertiary hospital with about 1,700 bed capacity. It receives referrals mostly from the southern sector of Ghana. The study was approved by the Ethical and Protocol Review Committee of the College of Health Science, University of Ghana (I.D: CHS-Et/M.7 -4.1/2018-2019). Permission to access the patients´ medical records was obtained from the medical records unit of the hospital but no informed consent was obtained because of the retrospective nature of the study. The diagnosis of HCC was based on any one of the following; histopathological confirmation of HCC, presence of a liver mass with characteristic HCC imaging changes on computed tomography (CT) or magnetic resonance imaging (MRI), alpha-fetoprotein (AFP) levels >165.2 IU/ML and liver mass on ultrasonography (USG) or clinical context (weight loss, hard nodular hepatomegaly or presence of a chronic liver disease or cirrhosis) positive serological markers of HBV or HCV infections and alpha-fetoprotein (AFP) levels >165.2 IU/ML [8, 9]. The following cases were excluded; medical records with incomplete data to confirm a diagnosis of HCC, persons less than 13 years as the GI Clinic only sees patients above 13 years and cases in which liver metastasis could not be excluded conclusively after review of the records. The following information was gathered from the medical records of patients meeting the inclusion criteria; demographic data, presenting symptoms, knowledge of and medical care for HBV status prior to diagnosis, laboratory results (HBsAg, Anti-HCV, HIV, LFT, FBC, AFP), results of various imaging modalities, liver histology if done, and treatment received. The data was anonymized and safely stored on a computer. Descriptive statistics was used for analysis of patients´ demographic information and other categorical variables. Measured outcomes were analyzed as mean±standard deviation. These were presented as tables. Associations and differences in categorical outcome variables were determined by the Chi-square test, while grouped sample t-test or the one-way analysis of variance (ANOVA) was used for continuous outcome measures at 95% confidence interval.

## Results

A total of 194 cases of HCC met the inclusion criteria and data extracted for analysis. There were 129 males and 65 females giving a male:female ratio of 2:1. The age of the cases ranged from 16-85 years with mean age of 45.2 years. The mean ages of males and females were 43.3 and 48.9 years respectively. Other demographic features of the cases are shown in Table 1; 136 (71.6%) of 190 were married, 39.2% admitted to alcohol intake and 6.7% to smoking. Fifty-two (43.3%) males and 15 (29.4%) females admitted to alcohol use whiles 9 (7.6%) males and 2 (4.4%) females admitted to smoking. Weight loss (77.1%) and abdominal pain (73.1%) were the major presenting symptoms. The other presenting symptoms are also shown in Table 1. HBV was the leading aetiological factor associated with HCC. HBsAg was positive in 104/145 (71.7%) of cases tested whiles 8 (8.5%) out of 94 and 2 (7.4%) out of 27 cases tested positive for HCV and HIV respectively. No patient had infection with more than 1 virus. HBsAg test was positive in 76.8% of males and 72.0% of females. There was significant association between HBsAg status and the age groups but no other demographic or clinical parameters (Table 1).

**Table 1:** Demographic, clinical and laboratory characteristics of hepatocellular patients according to HBsAg status

Variable	Variable	HBsAg status			Chi-square
	Total n (%)	Negative	Positive	Not indicated	(p-value)
Sex					0.66 (0.718)
Male	129 (66.5)	22 (61.1)	73 (67.0)	34 (69.4)	
Female	65 (33.5)	14 (38.9)	36 (33.0)	15 (30.6)	
Age group					21.8 (0.005)
<30	14 (7.5)	2 (6.1)	11 (10.2)	1 (2.1)	
30-39	60 (31.9)	7 (21.2)	41 (38.0)	12 (25.5)	
40-49	44 (23.4)	5 (15.2)	29 (26.9)	10 (21.3)	
50-59	43 (22.9)	9 (27.3)	20 (18.5)	14 (29.8)	
60+	27 (14.4)	10 (30.3)	7 (6.5)	10 (21.3)	
Marital status	190				1.78 (0.411)
Single	54 (28.4)	8 (22.9)	29 (27.1)	17 (35.4)	
Married	136 (71.6)	27 (77.1)	78 (72.9)	31 (64.6)	
Substance use					
Alcohol	67 (39.2)	8 (26.7)	38 (40.0)	21 (45.7)	2.81 (0.246)
Smoking	11 (6.7)	3 (11.5)	5 (5.5)	3 (6.4)	1.19 (0.551)
Symptoms	194				
Surveillance	0.	0	0	0	
Jaundice	87 (44.9)	12 (33.3)	54 (49.5)	21 (42.9)	2.97 (0.225)
Pedal swelling	59 (30.4)	11 (30.6)	32 (29.4)	16 (32.7)	0.17 (0.917)
Abdominal distention	83 (42.8)	10 (27.8)	49 (45.0)	24 (49.0)	4.28 (0.117)
Weight loss	147 (75.8)	30 (83.3)	78 (71.6)	39 (79.6)	2.56 (0.277)
Haematemesis	8 (4.1)	0 (0.0)	7 (6.4)	1 (2.0)	3.54 (0.170)
Meleana	8 (4.1)	1 (2.8)	6 (5.5)	1 (2.0)	1.23 (0.541)
Anorexia	88 (45.4)	15 (41.7)	48 (44.0)	25 (51.0)	0.91 (0.635)
Fever	17 (8.8)	3 (8.3)	11 (10.1)	3 (6.1)	0.67 (0.713)
Abdominal pain	144 (74.2)	25 (69.4)	83 (76.2)	36 (73.5)	0.66 (0.721)
Abdominal Mass/Lump	42 (21.7)	7 (19.4)	25 (22.9)	10 (20.4)	0.25 (0.881)
Poor sleep	52 (26.8)	10 (27.8)	28 (25.7)	14 (28.6)	0.16 (0.921)
Alpha Feto Protein level	141				6.44 (0.040)
<165.2U/ML	63 (44.7)	19 (65.5)	35 (38.9)	9 (40.9)	
≥165.2U/ML	78 (55.3)	10 (34.5)	55 (61.1)	13 (59.1)	
Hb Levels					8.80 (0.012)
Above reference	129 (66.5)	21 (58.3)	67 (61.5)	41 (83.7)	
AST					5.95 (0.051)
Above Normal	109 (76.2)	25 (78.1)	71 (80.7)	13 (56.5)	
ALT					4.99 (0.083)
Above Normal	96 (66.7)	20 (64.5)	65 (72.2)	11 (47.8)	

Sixty-five (62.5%) of 104 HBsAg positives were aware of their HBsAg status prior to the diagnosis of HCC. However, only 3 were receiving medical follow up and 2 were on treatment for HBV. The mean age for HBsAg positive HCC was less than for HCV and HIV; 41.7 years for HBV, 55.1 years for HCV, 43.5 years for HIV. The mean ages for HBsAg positives with concomitant alcohol use or smoking were 42.5 and 44.8 years respectively. The small numbers of HCV and HIV cases prevented evaluation of similar effect of alcohol on these viruses. The major laboratory test abnormality was AST elevations (76.2%) and low total protein (5.4%) the least frequent occurrence (Table 2). The alpha fetoprotein (AFP) results were recorded for only 141 patients and was abnormal (>10 IU/ML) in 81.6%. The AFP levels ranged from 0.54 to 750000 IU/ML with a mean AFP of 12866 IU/ML. More than half of the patients (52%) had AFP >165.3 IU/ML, the set cut off. The AFP and haemoglobin levels were significantly associated with HBsAg status (p = 0.040 and 0.012 respectively). One hundred and thirty-eight (138) of the 194 patients, had documented abdominal ultrasound examination and 50 abdominal CT scans but none had abdominal MRI. Liver histology was documented in 3 patients only. On abdominal ultrasound imaging, 15 (7.7%) had background cirrhosis, 44 (22.7%) presented with single liver mass, 78 (40.2%) with multiple liver masses and 41 (21.1%) with ascites. Analgesia was the first and only treatment prescribed to 144 of the patients and 4 patients were on sorafenib. One patient underwent microwave ablation. No patients in the cohort had undergone liver resection or transplant, or transarterial chemoembolization.

**Table 2:** Laboratory test results among hepatocellular patients

Variable	Test result			Total
	Below ref	Within ref	Above ref	
Lab test				
Hb	65 (33.5)	129 (66.5)	-	194
Platelet	24 (21.8)	86 (78.2)	-	110
T. Bilirubin	13 (9.5)	48 (35.0)	76 (55.5)	137
AST	-	34 (23.8)	109 (76.2)	143
ALT	-	48 (33.3)	96 (66.7)	144
T. Protein	7 (5.4)	96 (74.4)	26 (20.2)	129
Albumin	99 (70.2)	40 (28.4)	2 (1.4)	141

## Discussion

In this study, we have reported for the first time the demographic, clinical, laboratory and radiological characteristics of HCC from the KBTH, in Ghana. The demographic characteristics of HCC cases at KBTH appear to confirm previously known trends about the disease in Ghana and the West African Region. The male predominance seen in this report has been previously reported in Ghana, West Africa and globally [10-12]. This has previously been attributed to the higher prevalence of risk factors including chronic viral hepatitis, environmental carcinogens like alcohol and smoking, higher testosterone levels and lower levels of IL-6 production in males [13, 14]. Indeed in this study, higher prevalence of alcohol use and smoking was found in males than females. The relative importance of hepatitis to HCC causation varies greatly worldwide. This study confirms the important contribution of HBV to HCC in Ghana as in many other SSA countries [2, 12]. HBsAg positivity of 71.7% is higher than 52% previously reported in 2015 from Kumasi in Ghana and 60% from The Gambia and 48% from Ethiopia, both of which also have very high population prevalence of HBV [9, 11, 15]. The positive aspect to this finding is that HCC in Ghana remains a largely preventable cancer if the appropriate HBV control measures like childhood vaccination as in Taiwan and access to antiviral therapy can be effectively implemented [16, 17]. This study also highlights the unfortunate poor health seeking behavior of the cohort and possibly the Ghanaian population in general. Whilst almost two-thirds of the HBsAg positive had prior knowledge of their positive HBsAg status, their medical follow up and treatment for same was poor. This is likely attributable to the largely asymptomatic nature of chronic HBV infections until late, when complications set in. Other reasons could include seeking help from faith based care providers for reasons such as cost, greater satisfaction with care provided by these faith based healers [18]. Beyond screening to identify the infected population, vigorous education will be required to change this behaviour.

Hepatocellular carcinoma attributable to HBV from this study occurred at a relatively younger age than those attributable to HCV. This is similar to what has been reported from other HBV endemic regions of the world [3, 12, 19]. The reasons for this observation include the early age of infection with HBV in these regions. It is however surprising that alcohol use did not impact on the age of presentation of HCC especially as a synergistic effect of alcohol and HBV on HCC causation in some West African countries has been reported [20]. Umoh *et al*. however had earlier found alcohol to have only a minor role as a risk factor among HCC patients in The Gambia [9]. More work is needed to define the factors that drive the synergism between alcohol and HBV to cause HCC. The typical HCC patient from this study presented with abdominal pain and/or weight loss. This is very similar to a previous report among HCC patients in the Gambia and South Africa [9, 21]. The lack of patients identified through surveillance, the recommended way to diagnosing early HCC is consistent with global trends of underutilization of surveillance for early detection of HCC [22]. Factors that have been shown to affect utilization of surveillance include clinician knowledge and level of training as well as patient factors like myths that healthy diet and lack of symptoms are protective against HCC [23-25]. This study however did not assess the reasons for our findings. Abnormal AFP proportion reported in this study is similar to that reported (60-80%) in literature [26, 27]. The optimal cut off and use of AFP in diagnosis however remains controversial. At least in Asian patients with suspicious liver lesions, the cut-off AFP level of 200 ng/ml (165.2 IU/ML) is reported as useful to achieve a diagnosis of HCC with high specificity and reasonable sensitivity [8, 26]. More than 50% of patients in this study had AFP levels beyond this cut off. Despite its many limitations as a diagnostic tumour marker, it is difficult to discount AFP as a useful tool in resource poor economies like Ghana. More work is needed to establish appropriate cut off and acceptable sensitivity and specificity for diagnosis. The imaging modality for diagnosis of HCC in this study was limited to USG imaging in the majority of cases even though it is inferior to CT and MRI and recommended only as a cost effective screening tool by guidelines [8, 28-30]. The low use of these recommended multiphase imaging techniques is likely due to cost or non-availability. It is important that as a first step, ultra sonographers are properly trained to identify and characterize liver masses whiles efforts are made to improve the availability CT scans and MRIs. The proportion of patients in many African countries receiving specific HCC treatment has been disappointingly low compared to the trend in Asia, Europe and North America [12, 31]. Gyedu *et al*. have also previously reported that HCC patients in Ghana were not eligible for surgical resection [11]. This study similarly finds low utilization of potentially curative therapies of HCC like resection, radiofrequency ablation and percutaneous ethanol injection. The reasons as from previous reports are likely due to late presentation and with advanced diseases not amenable to these therapies, non-availability of these treatment modalities and the high cost of these treatments even when available. There is therefore a need to improve efforts aimed at early diagnosis and access to specific HCC treatment. This will require commitment from policy makers.

### Limitations

This was a retrospective review of medical records and therefore some aspects of the data collected were incomplete. This had the potential to create bias in the analysis and interpretation. Additionally, vital information like HBeAg status and HBV DNA levels were unavailable for majority of the patients. Also, KBTH is a referral centre, and so data from here may not be representative of the HCC burden and characteristics in other facilities across the country. Finally, it is likely that some cases that were referred to other specialists for other modalities of treatment like resection, microwave ablation and TACE, did not return to the unit and so their records were unavailable for analysis.

## Conclusion

HCC presenting to KBTH affects more males than females. Hepatitis B virus infection is the leading aetiologial risk factor associated with HCC. Most HBV infected patients were aware of their infection status but were not receiving medical care and/or treatment for HBV prior to HCC diagnosis. No patients were identified through surveillance. Presentations with the major symptoms of weight loss and abdominal pain qualifies most patients for only supportive rather than curative HCC treatment at KBTH. There is an urgent need to improve education of the population on hepatitis and its dreaded complication HCC. Health care providers should also be provided with HCC surveillance education and should establish programmes to implement same. Finally, policy makers should invest resources to improve diagnosis and treatment. If these are done, only then can we hope to identify early disease and offer specific or curative treatment to HCC patients.

### What is known about this topic

- Hepatocellular carcinoma is a leading cause of cancer deaths in Ghana;
- No HCC patients reporting to another tertiary facility in Ghana qualify for surgical interventions (resection or transplant).

### What this study adds

- No HCC patients presenting to KBTH were identified through surveillance;
- Most hepatitis B positive patients who develop HCC are aware of their infection status but do not get follow up or treatment for HBV prior to HCC diagnosis;
- Majority of HCC patients do not also qualify for curative non-surgical therapies.

## Competing interests

The authors declare no competing interests.
